# Supplementary material for: Effect of Intermittent Fasting on Cardiometabolic Health in the Chinese Population: A Meta-Analysis of Randomized Controlled Trials
Source: Nutrients. 2024 Jan 25;16(3):357. doi: 10.3390/nu16030357 (PMC10857210; doi:10.3390/nu16030357)
Supplement: Supplementary file 1 [file nutrients-16-00357-s001.zip › nutrients-2796938-supplementary.pdf]

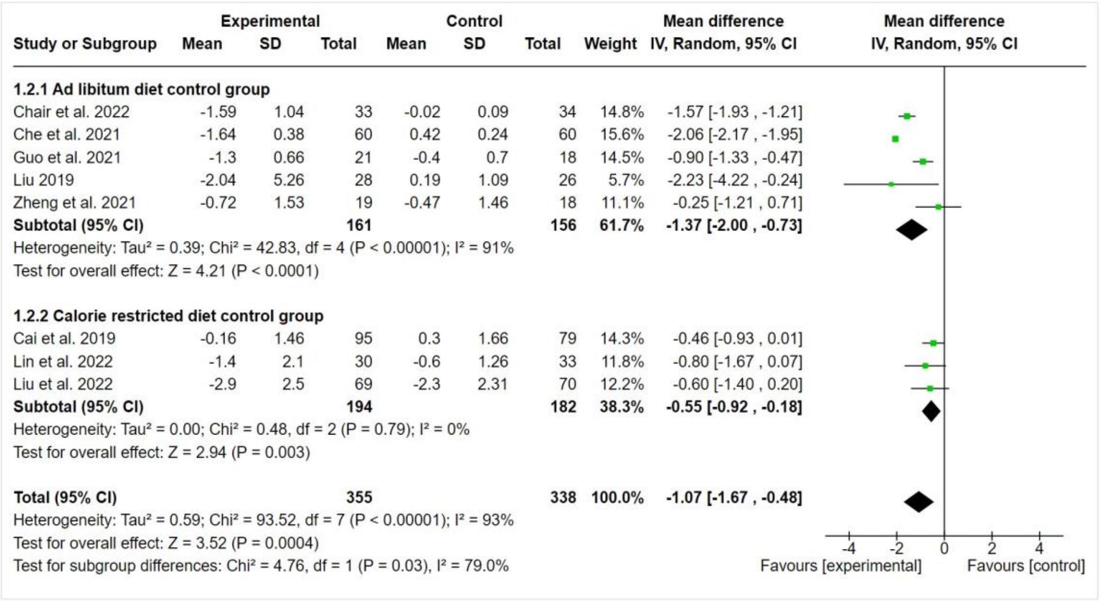


**Figure S1.** Forest plot for differences in body mass index (in kg/m$^2$) between intermittent fasting and control groups [12,17–24].

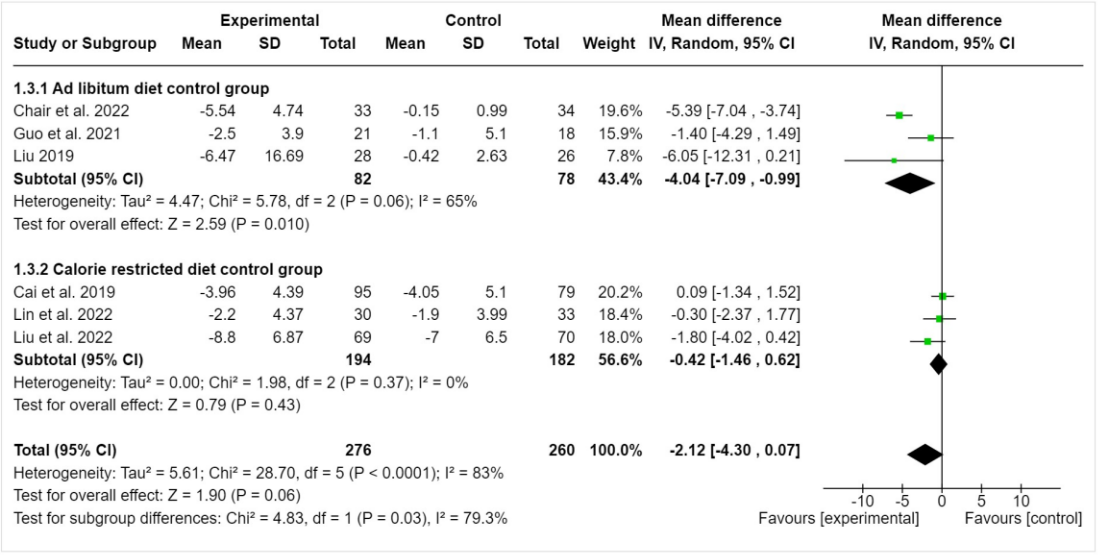


**Figure S2.** Forest plot for differences in waist circumference (in cm) between intermittent fasting and control groups [12,17,18,20–22].

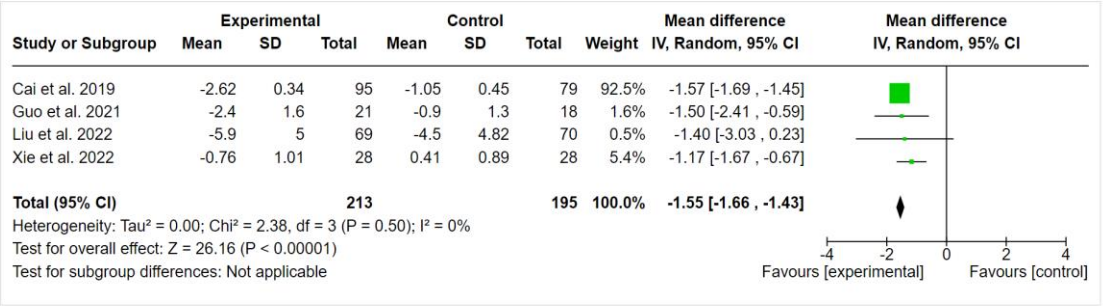


**Figure S3.** Forest plot for differences in body fat mass (in kg) between intermittent fasting and control groups [12,17,20,23].

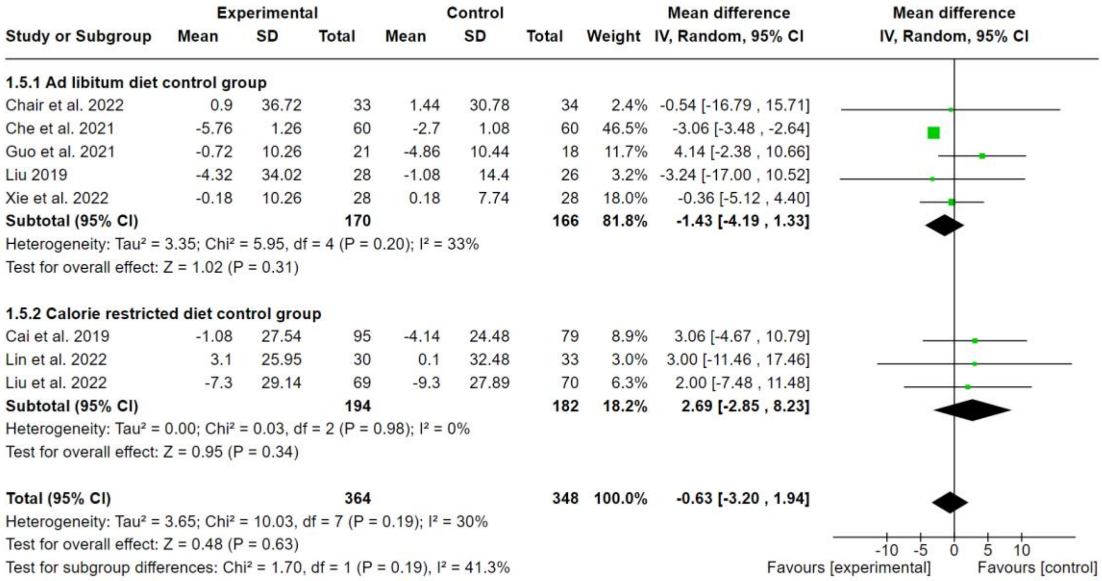


**Figure S4.** Forest plot for differences in total cholesterol (in mg/dL) between intermittent fasting and control groups [12,17–23].

| Study or Subgroup | Experimental Mean | Experimental SD | Experimental Total | Control Mean | Control SD | Control Total | Weight | Mean difference IV, Random, 95% CI |
|---|---|---|---|---|---|---|---|---|
| **1.6.1 Ad libitum diet control group** | | | | | | | | |
| Chair et al. 2022 | -1.44 | 12.24 | 33 | 2.34 | 8.28 | 34 | 2.8% | -3.78 [-8.80 , 1.24] |
| Che et al. 2021 | -2.88 | 0.72 | 60 | -2.7 | 0.9 | 60 | 43.7% | -0.18 [-0.47 , 0.11] |
| Guo et al. 2021 | 0.9 | 3.24 | 21 | 2.88 | 3.6 | 18 | 11.8% | -1.98 [-4.14 , 0.18] |
| Liu 2019 | 0.72 | 20.88 | 28 | 0.54 | 6.48 | 26 | 1.1% | 0.18 [-7.95 , 8.31] |
| Xie et al. 2022 | -0.54 | 3.6 | 28 | 0.9 | 3.06 | 28 | 15.9% | -1.44 [-3.19 , 0.31] |
| **Subtotal (95% CI)** | | | **170** | | | **166** | **75.4%** | **-0.92 [-2.00 , 0.15]** |
| Heterogeneity: Tau² = 0.54; Chi² = 6.36, df = 4 (P = 0.17); I² = 37% | | | | | | | | |
| Test for overall effect: Z = 1.68 (P = 0.09) | | | | | | | | |
| **1.6.2 Calorie restricted diet control group** | | | | | | | | |
| Cai et al. 2019 | -0.18 | 8.1 | 95 | 0.36 | 9.18 | 79 | 8.9% | -0.54 [-3.14 , 2.06] |
| Lin et al. 2022 | 1.5 | 6.7 | 30 | -0.2 | 5.28 | 33 | 7.0% | 1.70 [-1.30 , 4.70] |
| Liu et al. 2022 | 4.6 | 8.12 | 69 | 2.9 | 7.76 | 70 | 8.7% | 1.70 [-0.94 , 4.34] |
| **Subtotal (95% CI)** | | | **194** | | | **182** | **24.6%** | **0.88 [-0.70 , 2.45]** |
| Heterogeneity: Tau² = 0.00; Chi² = 1.80, df = 2 (P = 0.41); I² = 0% | | | | | | | | |
| Test for overall effect: Z = 1.09 (P = 0.28) | | | | | | | | |
| **Total (95% CI)** | | | **364** | | | **348** | **100.0%** | **-0.43 [-1.29 , 0.44]** |
| Heterogeneity: Tau² = 0.42; Chi² = 10.08, df = 7 (P = 0.18); I² = 31% | | | | | | | | |
| Test for overall effect: Z = 0.97 (P = 0.33) | | | | | | | | |
| Test for subgroup differences: Chi² = 3.41, df = 1 (P = 0.06), I² = 70.7% | | | | | | | | |

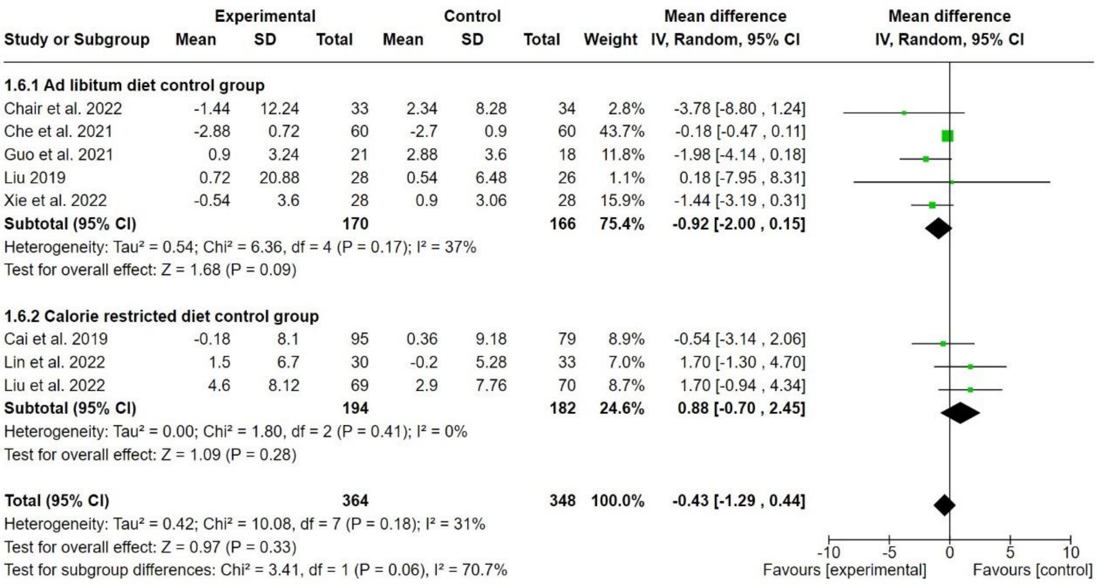


**Figure S5.** Forest plot for differences in high density lipoprotein cholesterol (in mg/dL) between intermittent fasting and control groups [12,17–23].

| Study or Subgroup | Experimental Mean | Experimental SD | Experimental Total | Control Mean | Control SD | Control Total | Weight | Mean difference IV, Random, 95% CI |
|---|---|---|---|---|---|---|---|---|
| **1.7.1 Ad libitum diet control group** | | | | | | | | |
| Chair et al. 2022 | -4.5 | 23.58 | 33 | -1.98 | 18.54 | 34 | 5.5% | -2.52 [-12.70 , 7.66] |
| Che et al. 2021 | -7.56 | 2.34 | 60 | -3.78 | 2.34 | 60 | 27.5% | -3.78 [-4.62 , -2.94] |
| Guo et al. 2021 | 0.36 | 6.3 | 21 | 9.9 | 11.34 | 18 | 11.8% | -9.54 [-15.43 , -3.65] |
| Liu 2019 | -7.2 | 15.12 | 28 | 1.62 | 13.32 | 26 | 8.5% | -8.82 [-16.41 , -1.23] |
| Xie et al. 2022 | 0 | 8.82 | 28 | -1.44 | 6.48 | 28 | 17.0% | 1.44 [-2.61 , 5.49] |
| **Subtotal (95% CI)** | | | **170** | | | **166** | **70.4%** | **-4.11 [-7.66 , -0.57]** |
| Heterogeneity: Tau² = 9.15; Chi² = 11.78, df = 4 (P = 0.02); I² = 66% | | | | | | | | |
| Test for overall effect: Z = 2.27 (P = 0.02) | | | | | | | | |
| **1.7.2 Calorie restricted diet control group** | | | | | | | | |
| Cai et al. 2019 | -0.36 | 15.66 | 95 | -0.72 | 13.68 | 79 | 16.0% | 0.36 [-4.00 , 4.72] |
| Lin et al. 2022 | -1.5 | 21.04 | 30 | 0.7 | 15.35 | 33 | 6.5% | -2.20 [-11.37 , 6.97] |
| Liu et al. 2022 | -8.4 | 26.23 | 69 | -8.9 | 25.79 | 70 | 7.1% | 0.50 [-8.15 , 9.15] |
| **Subtotal (95% CI)** | | | **194** | | | **182** | **29.6%** | **-0.01 [-3.59 , 3.58]** |
| Heterogeneity: Tau² = 0.00; Chi² = 0.26, df = 2 (P = 0.88); I² = 0% | | | | | | | | |
| Test for overall effect: Z = 0.00 (P = 1.00) | | | | | | | | |
| **Total (95% CI)** | | | **364** | | | **348** | **100.0%** | **-2.86 [-5.52 , -0.21]** |
| Heterogeneity: Tau² = 6.49; Chi² = 15.99, df = 7 (P = 0.03); I² = 56% | | | | | | | | |
| Test for overall effect: Z = 2.11 (P = 0.03) | | | | | | | | |
| Test for subgroup differences: Chi² = 2.55, df = 1 (P = 0.11), I² = 60.8% | | | | | | | | |

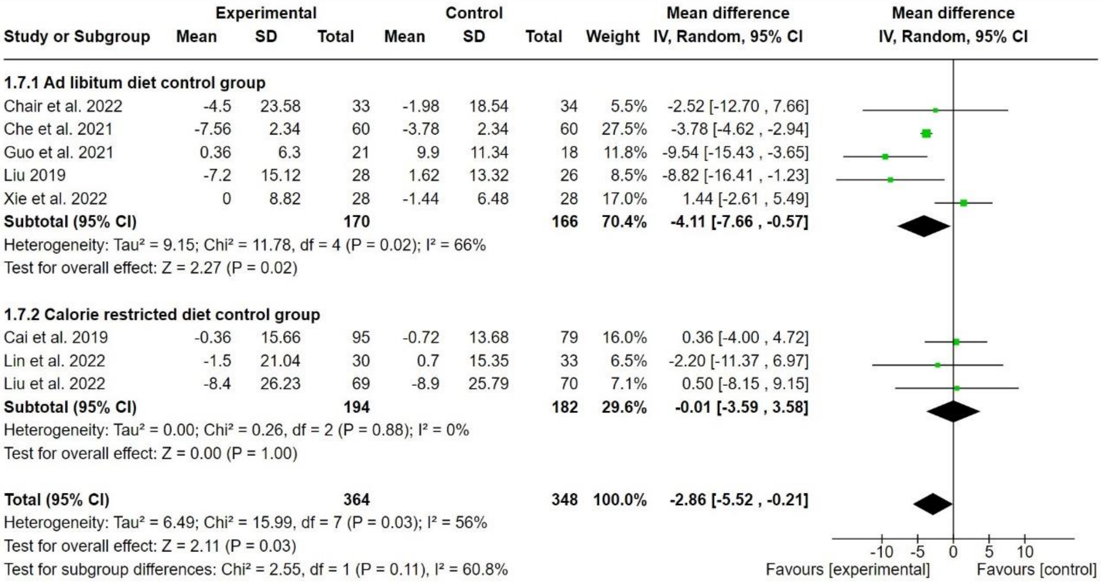


**Figure S6.** Forest plot for differences in low density lipoprotein cholesterol (in mg/dL) between intermittent fasting and control groups [12,17–23].

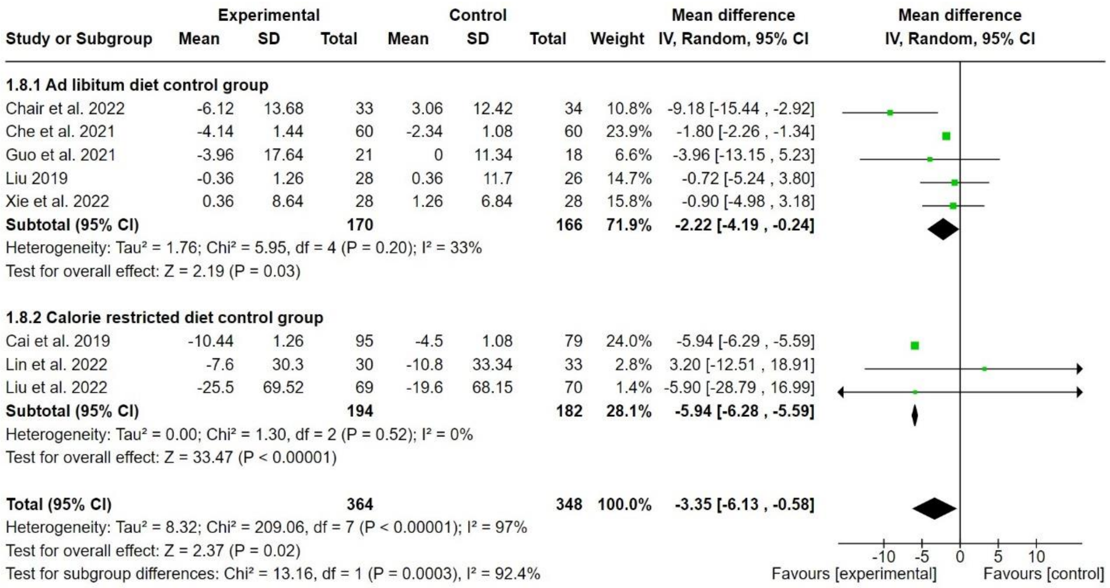


**Figure S7.** Forest plot for differences in triglycerides (in mg/dL) between intermittent fasting and control groups [12,17–23].

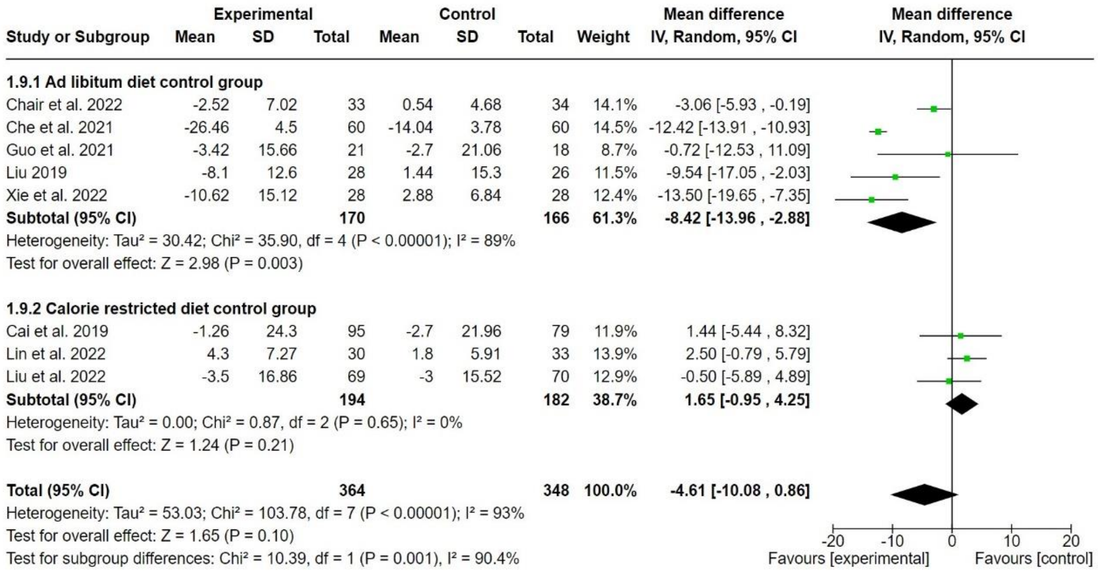


**Figure S8.** Forest plot for differences in fasting glucose or fasting plasma glucose (in mg/dL) between intermittent fasting and control groups [12,17–24].

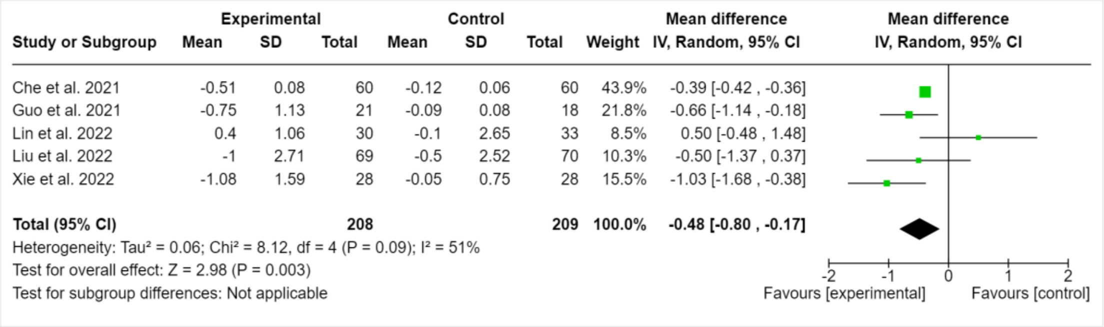


**Figure S9.** Forest plot for differences in homeostatic model assessment of insulin resistance between intermittent fasting and control groups [12,19–21,23].

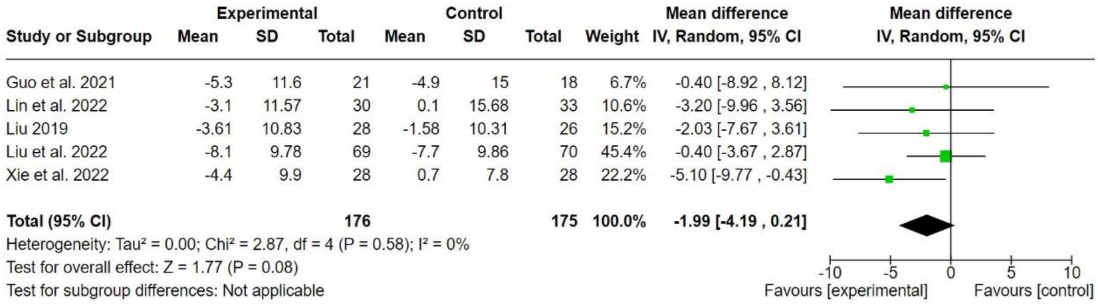


**Figure S10.** Forest plot for differences in systolic blood pressure (in mmHg) between intermittent fasting and control groups [12,20–23].

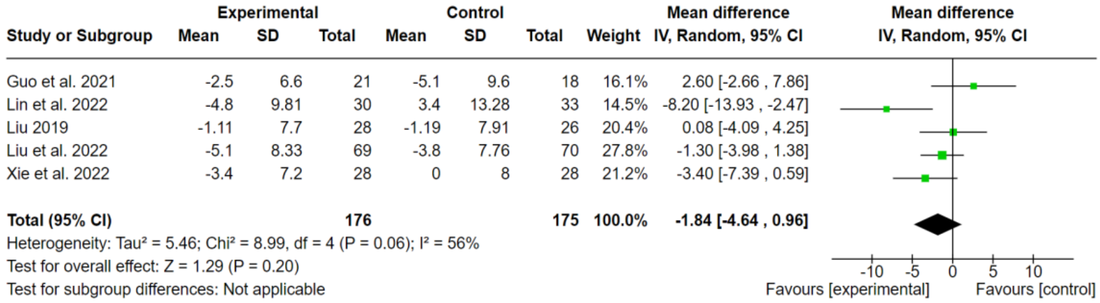


**Figure S11.** Forest plot for differences in diastolic blood pressure (in mmHg) between intermittent fasting and control groups [12,20–23].

**Table S1.** Search terms used in the literature review.

| **Databases** | **Search terms used** |
|---|---|
| Ovid platform:<br>1. Ovid MEDLINE<br>2. Embase (via Ovid)<br>3. Ovid Emcare | (intermittent fasting or time restricted feeding or periodic fasting) AND (randomized controlled trial or clinical trial or controlled clinical trial or randomization or random assignment) |
| China National Knowledge Infrastructure (CNKI) | (間歇性斷食) |
| WanFang | (intermittent fasting trial) |

**Table S2.** Reported adverse events of the included studies.

| Author, year | Reported adverse events (AEs) | Example of AEs (intervention group % vs control group %) (if applicable) |
|---|---|---|
| Cai et al., 2019 [17] | No severe AEs | -- |
| Chair et al., 2022 [18] | No severe AEs | -- |
| Che et al., 2021 [19] | No severe AEs | -- |
| Guo et al., 2021 [20] | No severe AEs | -- |
| Lin et al., 2022 [21] | Did not mention | -- |
| Liu 2019 [22] | Occurrence of mild AEs uncommon in both intervention and control groups<br>Overall (7.33% vs. 3.85%) | Decreased concentration (2.86% vs. 0.38%)<br>Thirsty (1.79% vs. 0.77%)<br>Dizziness (1.07% vs. 0.38%)<br>Low blood sugar level (0.54% vs. 0.19%)<br>Abdominal bloating (0.18% vs. 1.15%)<br>Constipation (0.36% vs. 0.58%)<br>Unstable emotion (0.54% vs. 0.38%) |
| Liu et al., 2022 [12] | Occurrence of mild AEs were similar in both intervention and control groups | Fatigue, dizziness, headache, decrease appetite, upper abdominal pain, dyspepsia and constipation |
| Xie et al., 2022 [23] | No severe AEs | -- |
| Zheng et al., 2021 [24] | Some AEs in both intervention and control groups | Hypoglycaemia (5.3% vs. 0%)<br>Hunger (5.3% vs. 0%)<br>Infection (5.3% vs. 5.5%)<br>Irritability (10.5% vs. 11.1%)<br>Nausea (5.3% vs. 0%) |

**Table S3**. Sensitivity analysis summary of the included studies in body weight and BMI.

| Study excluded | | Body weight | | | BMI | | |
|---|---|---|---|---|---|---|---|
| | | $I^2$ (%) | P for heterogeneity | Mean Difference [95% CI] | $I^2$ (%) | P for heterogeneity | Mean Difference [95% CI] |
| **Cai et al., 2019 [17]** | Overall | 68 | 0.002 | -2.44 [-3.10, -1.77] | 90 | <0.001 | -1.18 [-1.76, -0.60] |
| | ad libitum diet | -- | -- | -- | -- | -- | -- |
| | calorie-restricted diet | 0 | 0.71 | -1.41 [-2.97, 0.16] | 0 | 0.74 | -0.69 [-1.28, -0.10] |
| **Chair et al., 2022 [18]** | Overall | 83 | <0.001 | -1.88 [-2.37, -1.39] | 93 | <0.001 | -0.99 [-1.75, -0.23] |
| | ad libitum diet | 0 | 0.64 | -2.15 [-2.28, -2.01] | 92 | <0.001 | -1.29 [-2.25, -0.34] |
| | calorie-restricted diet | -- | -- | -- | -- | -- | -- |
| **Che et al., 2021 [19]** | Overall | 82 | <0.001 | -2.28 [-3.16, -1.41] | 70 | 0.003 | -0.88 [-1.33, -0.44] |
| | ad libitum diet | 78 | 0.001 | -2.87 [-4.15, -1.59] | 72 | 0.01 | -1.11 [-1.74, -0.49] |
| | calorie-restricted diet | -- | -- | -- | -- | -- | -- |
| **Guo et al., 2021 [20]** | Overall | 89 | <0.001 | -2.19 [-2.79, -1.60] | 92 | <0.001 | -1.10 [-1.75, -0.45] |
| | ad libitum diet | 81 | <0.001 | -2.73 [-3.71, -1.76] | 85 | <0.001 | -1.55 [-2.16, -0.93] |
| | calorie-restricted diet | -- | -- | -- | -- | -- | -- |
| **Lin et al., 2022 [21]** | Overall | 89 | <0.001 | -2.26 [-2.82, -1.70] | 93 | <0.001 | -1.11 [-1.75, -0.47] |
| | ad libitum diet | -- | -- | -- | -- | -- | -- |
| | calorie-restricted diet | 0 | 0.79 | -1.40 [-1.60, -1.21] | 0 | 0.77 | -0.50 [-0.90, -0.09] |
| **Liu, 2019 [22]** | Overall | 89 | <0.001 | -2.17 [-2.71, -1.62] | 94 | <0.001 | -1.00 [-1.62, -0.38] |
| | ad libitum diet | 79 | <0.001 | -2.55 [-3.30, -1.79] | 93 | <0.001 | -1.30 [-1.97, -0.62] |
| | calorie-restricted diet | -- | -- | -- | -- | -- | -- |
| **Liu et al., 2022 [12]** | Overall | 89 | <0.001 | -2.23 [-2.80, -1.67] | 93 | <0.001 | -1.14 [-1.77, -0.51] |
| | ad libitum diet | -- | -- | -- | -- | -- | -- |
| | calorie-restricted diet | 0 | 0.79 | -1.40 [-1.59, -1.20] | 0 | 0.5 | -0.54 [-0.95, -0.12] |
| **Xie et al., 2022 [23]** | Overall | 89 | <0.001 | -2.27 [-2.89, -1.66] | -- | -- | -- |
| | ad libitum diet | 80 | <0.001 | -2.90 [-4.03, -1.77] | -- | -- | -- |
| | calorie-restricted diet | -- | -- | -- | -- | -- | -- |
| **Zheng et al., 2021 [24]** | Overall | 89 | <0.001 | -2.22 [-2.78, -1.66] | 93 | <0.001 | -1.18 [-1.79, -0.56] |
| | ad libitum diet | 81 | <0.001 | -2.67 [-3.48, -1.86] | 90 | <0.001 | -1.59 [-2.21, -0.96] |
| | calorie-restricted diet | -- | -- | -- | -- | -- | -- |

**Table S4.** Pooled MDs when another intervention group in multi-arm studies was included.

| **Outcomes** | **MDs when ADF group in multi-arm studies was included** | **MDs when mTRE group in multi-arm studies was included** |
|---|---|---|
| **Body weight** | -2.16 [-2.27, -2.05], $I^2$ = 0% | -2.03 [-2.62, -1.44], $I^2$ = 89% |
| BMI | -0.84 [-1.61, -0.08], $I^2$ = 94% | -- |
| WC | -0.95 [-1.97, 0.08], $I^2$ = 7% | -- |
| **BFM** | -1.69 [-2.56, -0.82], $I^2$ = 89% | -1.30 [-1.81, -0.78], $I^2$ = 63% |
| TC | 0.19 [-2.72, 3.11], $I^2$ = 49% | 0.31 [-2.76, 3.38], $I^2$ = 49% |
| HDL | -0.52 [-1.55, 0.51], $I^2$ = 47% | -0.35 [-1.14, 0.43], $I^2$ = 24% |
| LDL | -2.59 [-5.29, 0.12], $I^2$ = 60% | -2.83 [-5.53, -0.13], $I^2$ = 58% |
| TG | 0.25 [-9.01, 9.50], $I^2$ = 100% | -3.02 [-5.79, -0.25], $I^2$ = 97% |
| FPG | -4.13 [-9.68, 1.41], $I^2$ = 100% | -4.38 [-9.93, 1.17], $I^2$ = 93% |
| HOMA-IR | -- | -0.16 [-0.60, 0.28], $I^2$ = 82% |
| SBP | -- | -2.22 [-4.46, 0.02], $I^2$ = 2% |
| DBP | -- | -2.16 [-5.16, 0.84], $I^2$ = 62% |

Abbreviations: ADF, alternate-day fasting; BMI, body mass index; BFM, body fat mass; DBP, diastolic blood pressure; FPG, fasting plasma glucose; HDL, high-density lipoprotein; HOMA-IR, homeostatic model assessment of insulin resistance; LDL, low-density lipoprotein; MDs, mean differences; mTRE, mid-day time restricted eating; TC, total cholesterol; TG, triglycerides; SBP, systolic blood pressure; WC, waist circumference.
